# Amphiphilic Bioactives of Freshwater Aquatic Plants *Nelumbo nucifera* (Indian Lotus) and *Lemna* sp. with Antioxidant, Anti-Inflammatory and Antithrombotic Activities: In Vitro Study

**DOI:** 10.3390/ph18060835

**Published:** 2025-06-02

**Authors:** Marina Seferli, Melina Lefkaki, Vasileios Manousakis, Anna Ofrydopoulou, Katie Shiels, Sushanta Kumar Saha, Grigorios Krey, Nikolaos Kamidis, Nikolaos Stamatis, Chryssa Anastasiadou, Alexandros Tsoupras

**Affiliations:** 1 Hephaestus Laboratory, Department of Chemistry, International Hellenic University, Kavala University Campus, 65404 Kavala, Greece; masmfer@chem.duth.gr (M.S.); lefkakimelina@gmail.com (M.L.); vamanvu@chem.duth.gr (V.M.); anofrid@chem.duth.gr (A.O.); 2 Centre for Applied Bioscience Research, Technological University of the Shannon—Midlands Midwest, Moylish Park, V94 E8YF Limerick, Ireland; katie.shiels@tus.ie (K.S.); sushanta.saha@tus.ie (S.K.S.); 3 Fisheries Research Institute, Hellenic Agricultural Organization-DIMITRA (ELGO-DIMITRA), Nea Peramos, 64007 Kavala, Greece; krey@elgo.gr (G.K.); nikkami@elgo.gr (N.K.); nikstam@elgo.gr (N.S.); anastasiadou@elgo.gr (C.A.)

**Keywords:** aquatic plants, *Nelumbo nucifera*, *Lemna*, antioxidants, antithrombotic, anti-inflammatory, platelet, PAF, ADP, omega-3s

## Abstract

**Background-Objectives:** Chronic diseases linked to inflammation, such as cardiovascular disease (CVD) and cancer, continue to pose major public health challenges due to their high mortality rates. There is growing interest in natural bioactive compounds, particularly those derived from plants, as potential therapeutic or preventive agents due to their low toxicity profiles. This study aimed to explore two freshwater plants—*Nelumbo nucifera* (Indian lotus) and *Lemna* sp.—as potential sources of bioactive compounds with antioxidant, anti-inflammatory, and antithrombotic properties. While *N. nucifera* has established but incompletely characterized biofunctional properties, *Lemna* sp. remains largely unexplored in this context. **Methods:** Amphiphilic extracts from both plant species were analyzed for phenolic and lipid constituents, including unsaturated fatty acids, polar lipids, and carotenoids. Antioxidant capacity was evaluated using DPPH, ABTS, and FRAP assays. Anti-inflammatory and antithrombotic activities were assessed via platelet aggregation assays using PAF and ADP agonists. Structural characterization was performed using **Fourier transform infrared spectroscopy** (FT-IR) and liquid chromatography–mass spectroscopy (LC-MS) to support structure–activity relationship (SAR) analysis. **Results:** Extracts, particularly from *Lemna* sp., showed potent antiplatelet activity against PAF and ADP. LC-MS revealed the presence of polar lipids rich in monounsaturated and omega-3 polyunsaturated fatty acids, with a favorable omega-6/omega-3 ratio, especially in *Lemna* sp., correlating with strong anti-inflammatory potential. High levels of total phenolics and carotenoids were observed, aligning with substantial antioxidant capacity in both species. **Conclusions:** These findings suggest that *N. nucifera* and *Lemna* sp. are promising sources of bioactive compounds with potential applications in functional foods, cosmetics, and pharmaceuticals targeting inflammation- and thrombosis-related chronic diseases. Further studies are warranted to confirm their safety and efficacy.

## 1. Introduction

Although modern medicine, chemistry and biology are advancing rapidly and life expectancy has increased significantly over previous years, certain chronic diseases inextricably linked to inflammation and thrombosis still constitute an important concern with respect to human health and wellbeing. These diseases, including diabetes, various cardiovascular diseases, chronic respiratory diseases and cancer, increase the mortality rate while, at the same time, imposing a burden on the lives of patients and their families, as well as on the healthcare system. Therefore, it could be said that they are a burden on the economy of countries globally [1]. The risk factors for these diseases are divided into variable and non-variable factors. Non-variable factors include gender, age, race and genetic profile, while variable factors include lifestyle (smoking, diet, physical activity, etc.) [1,2].

The presence and coexistence of key risk factors appear to activate underlying molecular and cellular pathways that drive the persistent development of chronic inflammation, subsequently leading to thrombo-inflammatory complications associated with chronic diseases. These conditions often evolve gradually, with pathogenic processes occurring well before overt cellular dysfunction and tissue damage become apparent. In the absence of adequate immune surveillance and preventive homeostatic mechanisms, disease progression becomes inevitable. In such cases, targeted medical intervention becomes essential to mitigate the elevated risk of morbidity and mortality. Conversely, the adoption of healthy lifestyle practices—including a balanced diet and regular physical activity—alongside the consumption of functional products with anti-inflammatory and antioxidant properties, has been shown to play a significant role in the prevention of chronic diseases by reducing chronic inflammation, oxidative stress, and thrombotic risk [1,2].

Research has shown that many natural sources contain a wealth of bioactive compounds with anti-inflammatory, antioxidant and antithrombotic activities [1,2,3]. A typical category of these sources includes various aquatic plants, which are an excellent source of compounds with recognized biological and therapeutic properties against these diseases. A wealth of active biological metabolites have been isolated from aquatic plants and used as colorants, additives, nutrients, and biofunctional ingredients that have contributed to new product discovery and drug development [4].

Indian lotus (*N. nucifera*) is one of the best known freshwater plants that contain compounds with biological and therapeutic properties. However, some studies have also reported that other more obscure aquatic plants may also contribute to the above approaches, such as members of the Araceae family (e.g., *Lemna* sp.) [5]. These floating aquatic plants grow in thick layers in still or slow-moving waters that are rich in nutrients [6] and in slow-moving rivers up to 2 m deep.

Lotus (*N. nucifera Gaertn*.) is a widely distributed aquatic plant in Asia, America and Australia. The flowers, rhizomes, and seeds of lotus have nutritional and medicinal uses, with the seeds being rich in carbohydrates, proteins, lipids, and polyphenols [7]. This plant is often cultivated for its elegant and sweet-scented flowers, making it the national flower of India [8]. However, the plethora of bioactive components present in this plant and their potential applications in various functional products have not been fully explored and require further analysis.

In addition to its growth in freshwater rivers and lakes, *Lemna* sp. has recently been observed to also grow on the surfaces of water catchments of urban water treatment plants rich in nitrogen and phosphorus [6,9]. Therefore, the continued growth and sustained availability of *Lemna* sp. in such aquatic facilities provides a potentially rich source of bioactive compounds with applications in chronic disease prevention [10].

The present study details the quantification and structural elucidation of the amphiphilic bioactive compounds with anti-inflammatory and antioxidant health-promoting properties present in both *N. nucifera* and *Lemna* sp.

## 2. Results and Discussion

### 2.1. Evaluation of the Extraction Efficiency for the Different Samples

The yields of extracts in terms of total lipids (TL), total amphiphilic compounds (TAC) and total lipophilic compounds (TLC) from different samples of the two aquatic plants are presented in Table 1 and expressed as a percentage for each sample (g of lipids extracted per g of sample). A modified version of the extraction procedure of Bligh and Dyer [11], combined with the countercurrent partitioning technique of Galanos and Kapula [12] according to Tsoupras et al. [13], was chosen in the present study for the extraction and separation of TL, TAC and TLC from whole plant samples (rhizome, flower, foliage and stem). The combination of these two methods allows for a simple and efficient extraction and separation approach to obtain bioactive dietary polar lipid components, as previously demonstrated by testing on several solid and liquid, plant-derived natural sources [14,15]. It also results in an excellent extraction efficiency for TAC bioactives from each of these natural sources while preserving their fatty acid composition. Therefore, it ensures very little to no loss of bioactive properties of biofunctional lipids during their extraction, unlike other methods, such as Soxhlet extraction, which uses high temperatures and, thus, may negatively affect the fatty acid content of the extracted lipids [16].

*Lemna* sp. and *N. nucifera* (LM and NN, respectively) showed similar results in terms of percentages of extracted TAC, TLC and TL.

In all analyzed LM and NN samples, TAC constituted about 64% of the TL, while the remaining 33% of the TL appeared to be TLC content, suggesting that all LM and NN samples contained higher amounts of TAC than TLC.

Thus, even though a lower yield in TL was observed in comparison to previous studies [17], considerable amounts of TAC were successfully extracted—results comparable to those of other studies. For example, in both LM and NN samples, TAC constituted about 64% of the TL, while the remaining 36% of the TL appeared to be TLC content, suggesting that they contain higher amounts of TAC than TLC.

Nevertheless, based on the analyses carried out on both seeds [7] and leaves [18] of lotus, it appears that, in the present study, the results were slightly lower than expected, which may be due to the amount of plant extracted and the fact that the analysis was carried out when the plant was fresh and no drying process was followed.

### 2.2. Analysis of the Anti-Inflammatory and Antithrombotic Activity of Extracted Polar Lipids and Neutral Fats

**Figure 1:**
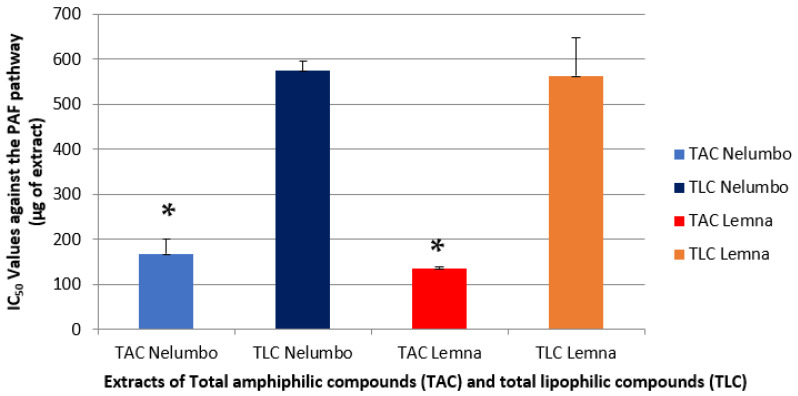
Schematic representation of the anti-inflammatory activity of *Lemna* sp. and *N. nucifera* TAC and TLC extracts against thrombotic and inflammatory mediator PAF. Results are expressed as mean IC_50_ values (μg of extract in the aggregometer cuvette containing 0.25 mL of human plasma rich in platelets), which are the concentration/amount of TAC/TLC that shows half maximum (50%) of anti-inflammatory potency against PAF-induced hPRP platelet aggregation. (* indicates a statistically significant difference (*p* < 0.05) between the anti-inflammatory IC_50_ values of TAC and TLC within each plant against the PAF pathway).

**Figure 2:**
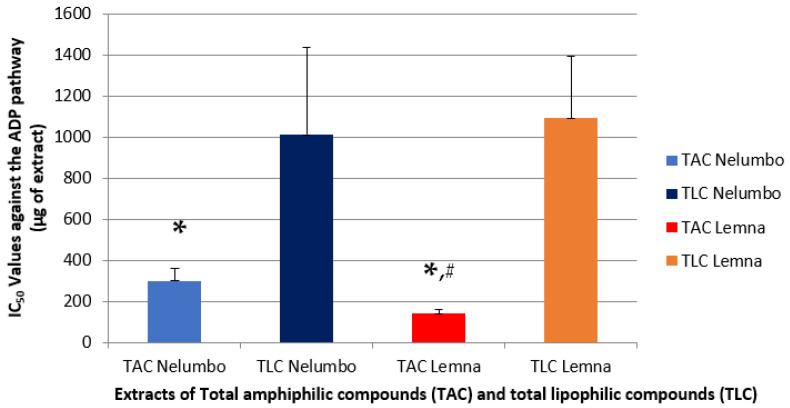
Schematic representation of the anti-platelet effects of *Lemna* sp. and *N. nucifera* TAC and TLC extracts against standard platelet agonist ADP. Results are expressed as mean IC_50_ values (μg of extract in the aggregometer cuvette containing 0.25 mL of human plasma rich in platelets), which are the concentration/amount of TAC/TLC that shows half maximum (50%) of anti-platelet potency against ADP-induced hPRP platelet aggregation. (* indicates a statistically significant difference (*p* < 0.05) between the anti-platelet IC_50_ values of TAC and TLC within each plant against the ADP-pathway; # indicates statistically a significant difference (*p* < 0.05) between the IC_50_ values of TAC from *Lemna* sp. and the relevant IC_50_ values of the TAC extracts from *N. nucifera* concerning their anti-ADP activities).

The biological activities of all extracts were evaluated by assessing their anti-inflammatory and antithrombotic potency against the activation and aggregation of human platelets induced by the inflammatory and thrombotic mediator, platelet activating factor (PAF) (Figure 1), as well as by the classical standard platelet agonist, Adenosine 5′ diphosphate (ADP) (Figure 2), as previously described [13]. The results are expressed as IC_50_ values (mean of μg of extract in the aggregometer cuvette containing 0.25 mL of human plasma rich in platelets), meaning their concentration/amount that shows half maximum (50%) of anti-inflammatory/anti-platelet potency against the aggregation of the PAF/ADP pathway of human plasma rich in platelets (hPRP). It should be noted that the lower the IC_50_ value for an extract, the greater its inhibitory effect against the given platelet-aggregating inflammatory/thrombotic agonist.

As suggested by the results presented in Figure 1, the anti-inflammatory activity of the bioactive extracts from these plants against the PAF pathway seems to be mainly derived from their amphiphilic compounds, since the TAC extracts showed statistically significantly much lower IC_50_ values and, thus, greater inhibitory effects against PAF compared to the corresponding lipophilic components (TLC) of each plant (Figure 1). When the two plants are compared, it appears that the TAC samples from *Lemna* sp. have an anti-PAF IC_50_ value similar to that of Indian lotus. However, for both plants, the TLC samples do not show any significant inhibitory activity in terms of inhibition of thrombotic and inflammatory mediator PAF (Figure 1). The finding that TAC extracts from both plants possess strong anti-PAF activities is very important and suggests further pharmacological uses for these bioactives, since PAF and its inflammatory pathway are implicated in several inflammation-related chronic disorders. Targeting PAF and its signaling pathways seems to be a promising route for pharmaceutical actions for both prevention and therapy [1,2,3].

Similar conclusions can be drawn about the activity of the extracts from both plants against the ADP agent (Figure 2). TAC extracts showed statistically significantly much lower IC_50_ values compared to the corresponding lipophilic components (TLC) of each plant (Figure 2). Nevertheless, *Lemna* sp.-derived TAC showed the lowest IC_50_ value against the ADP pathway and, therefore, higher activity against this platelet agonist.

These results show that the anti-inflammatory and antithrombotic activity of these two aquatic plants, both against the PAF pathway involved in inflammation and thrombosis and the ADP pathway involved in platelet aggregation, can be attributed to their amphiphilic components. More specifically, it appears that the amphiphilic components of *Lemna* sp. TAC extract are more effective against the ADP pathway (IC_50_ values ranging between approximately 120 and 160 μg of TAC) than the corresponding TAC of *N. nucifera*, which showed IC_50_ values ranging between approximately 240 and 360 μg of TAC.

Moreover, this anti-ADP activity of the *L.* TAC extracts was found to be similar to their activity against the PAF pathway, suggesting that the *Lemna* sp. TAC extracts possess similarly potent anti-inflammatory and anti-platelet activities against these two major thrombo-inflammatory pathways of platelet activation.

Interestingly, in the case of *N. nucifera*, the TAC exhibited statistically significantly lower IC_50_ values (*p* < 0.05) against the PAF pathway (IC_50_ values of approximately 130–200 μg of TAC) than against the ADP pathway (IC_50_ values of approximately 240–360 μg of TAC). These results suggest that *N. nucifera* TAC extracts have greater anti-inflammatory specificity against the PAF thrombo-inflammatory pathways of platelet activation than antiplatelets against ADP.

In previous relevant studies, *N. nucifera* extracts from flowers of various colors [19], as well as from the fruit [20], were found to be effective against the ADP factor. Furthermore, the results of the present study concerning the anti-PAF pathway are similar to the reported IC_50_ values from several Malaysian medicinal plants [21]. According to the above results, as well as studies on tea and nettle that have demonstrated anti-inflammatory and antithrombotic activity, a range of acceptable IC_50_ values can be considered against PAF and ADP for extracts from medicinal plants and/or herbs These values depend directly on the solvent used during extraction, as well as on the nature of the sample (fresh or dried) [22].

### 2.3. FT-IR Analysis for Identification of Classes of Bioactive Compounds Present in the TAC Extracts of Both Plants

**Figure 3:**
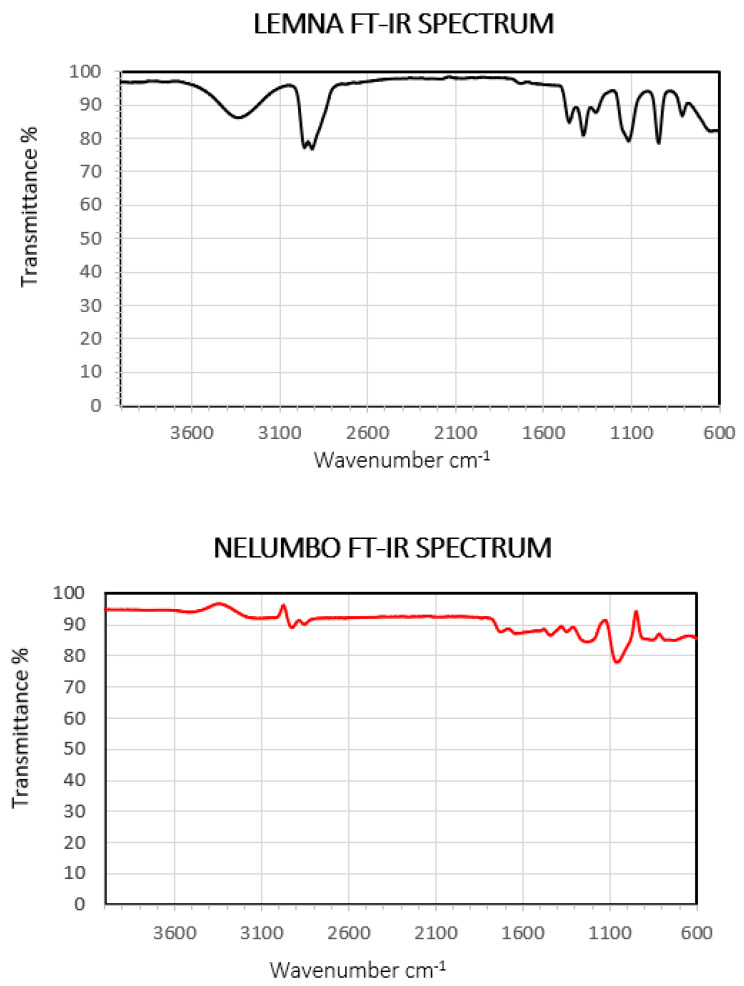
FT-IR spectra of *Lemna* sp. and *N. nucifera*.

Since the TAC extracts were highly bioactive against both inflammatory mediator PAF and platelet agonist ADP, ATR-FT-IR was utilized for an initial structural evolution of the molecular classes of the bioactive compounds present in the TAC of both LM and NN (Figure 3).

Table 2 presents the interpretation of the FT-IR peaks for *Lemna* sp., showing the bond vibrations to which the depicted absorptions correspond, as well as the similarities of the peaks with those of known compounds (standards).

In this comparison, structural elucidation of the TAC extracts showed that different types of bioactive phenolic compounds were present in these extracts, as specific functionable groups of hydrolysable tannins, such as gallic acid and flavonoids including catechin and quercetin were identified according to characteristic fingerprint peaks. Moreover, in the TAC extracts of both plants, characteristic peaks were identified for functional groups of bioactive polar lipids and carotenoids. Overall, this aquatic plant contains bioactive compounds with strong antioxidant power and potentially health-promoting properties against oxidative stress and related disorders. This conclusion is in agreement with previous studies [23] that obtained similar results for the investigated functional groups.

The FT-IR results related to *N. nucifera* are presented in Table 3.

The molecular classes present in the extract are similar to those from *LM*. Therefore, phenolic and carotenoid compounds and polar lipids of great biological importance were also detected in this case. However, the present spectrum seems to be more enriched than those found in the literature, which is probably due to the fact that the whole plant was used and not only a part of it, such as the flower [24] or the leaves [25], as has been the case in previous studies.

Phenolic compounds with functional groups like those present in flavonoids, including catechin and quercetin, as well as in carotenoids, have been proposed to provide many health benefits, including antioxidant, antimicrobial, anti-inflammatory, antiviral, cardioprotective and anticancer properties, as well as preventative and therapeutic applications in several diseases [26,27,28].

Polar lipids (PLs) are another class of bioactives identified to be present in the TAC extracts of both plants, as per the FT-IR outcomes shown in Table 2 and Table 3. Such PL bioactives have been associated with several health conditions, while those containing specific fatty acid contents esterified within their structures are of great importance as modulators of inflammatory conditions, thrombosis, and associated manifestations and disorders. Subsequently, such important PL bioactives need further attention, and more sophisticated analysis needs to be performed both to evaluate their fatty acid contents and to elucidate their overall structures in order to be able to make solid remarks about their contribution to the health-promoting effects observed in the present study. As ATR-FT-IR cannot provide such details for the structures of the identified PLs, further LC-MS analysis of these PL bioactives was, indeed, performed, as described below.

### 2.4. Analysis of the Fatty Acid Composition of the TAC Extracts from the Plants

In order to evaluate the fatty acid composition of the TAC extracts of the two studied aquatic plants, LC-MS analysis was performed. The aim was to determine both the fatty acids esterified in polar lipids and the contents of free, non-esterified fatty acids, as previously described [29]. These results are presented in Table 4 and Table 5.

The PLs of the TAC extracts from both plants were found to be rich in polyunsaturated fatty acids (PUFAs), with smaller amounts of saturated fatty acids (SFAs) and monounsaturated fatty acids (MUFAs) being the least abundant in the PL fraction. More specifically, the PL bioactives of the TAC extracts from *Lemna* sp. contained high amounts of essential omega-3 (n-3) PUFA α-linolenic acid (ALA, C18:3 c9,12,15 n-3), as well as essential omega-6 (n-6) PUFA linoleic acid (LA, C18: 2 c9, 12 n-6). Most importantly, the PLs of the TAC extracts from LM were found to contain significant amounts of highly unsaturated n-3 PUFAs eicosapentaenoic acid (EPA, C20:5 c5,8,11,14,17 n-3) and docosahexaenoic acid (DHA, C22:6 c4,7,10,13,16,19 n-3), which were detected only in low amounts in NN and are hardly detected in other land-based medicinal plants. Moreover, in the PLs of the TAC extracts from both *LM* and NN, palmitic acid (C16:0) was the most abundant SFA, while oleic acid (C18:1 c9) was the most abundant MUFA. These values are comparable to those of previous studies of these plants or plants of the same genus [30], with minor numerical differences due to differences in the sample mass and/or parts of the plant used for each analysis but mainly due to the type of the extracts (here, we analyze PLs from TAC, whereas in other studies, the total fatty acid composition was analyzed). More specifically, the differences observed in the fatty acid profiles between this study and previous studies may also be due to the fact that only the PLs from the TAC extracts were used in our analysis, as compared to the TL that was used in the other studies.

Independently of the observed differences, it is also worth mentioning that the presence of these essential n-3 PUFAs (not only that of ALA but mainly the significant amounts of EPA and DHA detected) bound (esterified) in the PLs of the TAC extracts further supports the anti-inflammatory potency observed for these TAC extracts, simultaneously providing a structure activity-based scientific explanation for their potent anti-inflammatory effects against the PAF pathway, which has also been previously thoroughly described for PLs in TAC extracts from other sources that are rich in MUFAs and n-3 PUFAs [1,2,3,13,14,15,29,31].

It has been shown that TAC extracts rich in oleic acid and/or n-3 PUFAs strongly inhibit mediators of inflammation like PAF in several tissues and cells, as well as in platelets, where, apart from their anti-PAF effects, they also show potent antithrombotic effects against thrombin and other standard platelet agonists like collagen and ADP [1,2,3,13,14,15,29,31]. Moreover, apart from the direct and/or indirect effects of these PL bioactives on the cell membrane receptors of such thrombo-inflammatory mediators, when these PLs are incorporated into cell membranes, their MUFA and/or n-3 PUFA contents are susceptible to specific cytoplasmic phospholipase A2 (cPLA2) enzymatic activity, resulting in their release in the cytoplasm, where they may exert different biological activities and anti-inflammatory health benefits. For example, the release of n-3 PUFA into the cytosol facilitates the production of anti-inflammatory eicosanoids that inhibit cyclo-oxygenases (COXs) and act antagonistically toward other inflammatory and thrombotic eicosanoids (prostaglandins, leukotrienes and thromboxanes). The precursors of these eicosanoids are n-6 PUFAs (e.g., arachidonic and linoleic acid), while n-3 PUFAs are also involved in the production of resolvins that promote the termination of the inflammatory response [31,32].

Another indicator that can be used as a measure of lipid anti-inflammatory potential is the n-6/n-3 PUFA ratio, for which it has been suggested that the lower its value, the better the preventive anti-inflammatory benefits against various chronic diseases related to inflammation and platelet accumulation [32]. Based on the data presented in Table 4, it could be said that the PLs in the TAC extracts from both plants have similar low n-6/n-3 values (0.3–0.4), further supporting their significant anti-inflammatory and anti-thrombotic potential.

At the same time, this effect may be enhanced by the smaller but appreciable amounts of n-3 PUFAs from the free fatty acids found in TAC extracts (Table 5), which provide an indicative value for the n-6/n-3 PUFA ratio, which is, nevertheless, also quite low in this type of fatty acid (non-esterified fatty acids).

More specifically, with respect to the content of the free non-esterified fatty acids in the TAC extracts, they were found to be rich in SFAs, while the amounts of PUFAs and MUFAs detected were relatively low. When comparing the plants, LM shows a higher amount of PUFAs, while Indian lotus stands out for its MUFA and SFA contents. The main SFAs detected in higher amounts in both plants are palmitic acid (C16:0) and stearic acid (C18:0). The main n-6 PUFAs—of course, in much lower amounts than the esterified fatty acids—is LA for both plants. The highest concentration of n-3 PUFAs was found in ALA, while the MUFA with the highest value seems to be oleic acid (C18:1 c9). Interestingly, even though the TAC extracts from *Lemna* sp. possess higher amounts of free PUFAs in comparison to the TAC extracts of *N. nucifera*, in both aquatic plants, the TAC extracts contained a relatively low and, thus, beneficial ratio of n-6/n-3 PUFAs, with similar values ranging around 1.0.

Ιt is worth noting that when comparing the two tables for each plant separately, it appears that in *NN*, most of the fatty acids are esterified to polar lipids and, thus, several are not detected in the analysis for free fatty acids. In *LM*, this phenomenon is less pronounced, as many of the fatty acids are present in small amounts in free form. At the same time, some fatty acids, such as C13:0, C20:0 and n6 PUFA C22:4 c7,10,13,16, are only detected as free acids in *Lemna* sp.

These findings suggest that the free fatty acids detected in the TAC extracts are not derived by lipolysis of the PLs present in the TAC. Instead, they seem to represent the free fatty acid contents of these plants at the time that the extractions took place; because they are less lipophilic than the triglycerides, they migrated into the TAC fraction, where they were detected, rather than into the TLC fraction of these extracts.

### 2.5. LC-MS Analysis and Structural Elucidation of PL Bioactives Present in the TAC Extracts of Freshwater Aquatic Plants N. nucifera and Lemna sp.

LC-MS was used to also examine the TAC extracts of *Lemna* sp. and *N. nucifera* to identify the classes of molecular polar lipid species of significance, with results shown in Table 6. Several PL classes were successfully identified by the LC-MS analysis of the TAC extracts. The identified were mainly glycerophospholipids (GPs), such as phosphatidylcholines (PCs), phosphatidylethanolamines (PEs), phosphatidic acids (PAs), phosphatidylinositols (PIs), phosphatidylserine (PS), phosphoglycerols (PGs); glyceroglycolipids (GLs) like monogalactosyldiacylgylcerols (DGDGs) and sulfoquinovosyldiacylglycerols (SQDGs); and several sphingolipids (SP)—specifically, ceramides (Cers), PI-ceramides (IPCs), hexosylceramides (HexCers), sulfatides (SHexCers), Mannosyl-PI-ceramides (MIPCs), sphingomyelins (SMs), PE-ceramides (CerPEs), ceramide phosphates (CerPs)—similarly to what has been reported to the literature for the PLs from these two aquatic plants [5].

GPLs, or glycerophospholipids, are essential parts of cell membranes. They are the main source of signaling molecules, including their long-chain PUFA and MUFA contents [33]. Phosphatidylcholine (PC), phosphatidylethanolamine (PE), phosphatidylserine (PS), and phosphatidylinositol (PI) are the primary components of membrane GPLs. The polar lipids of the extracts contained both diacyl lipids and alkyl–acyl lipids from each category of glycerolipids and sphingolipids, according to survey scans in the negative-ion mode between 500 and 1000 *m*/*z* for the LC-MS structural analysis of the glycerolipids and sphingolipids of both plants. Moreover, both oleic acid and n-3 PUFAs like ALA and EPA or DHA were observed to mainly be incorporated into the sn-2 glycerol/sphingosin backbone of all these GPLs and SPLs. The main characteristic of the PLs from both the *Lemna* sp. and the *N. nucifera* TAC extracts is their high UFA content (67%), with n3 PUFAs (mainly ALA but also EPA and DHA) reaching up to the 42% and the n6 PUFAs (mainly LA) accounting for up to 13–16%, while MUFAs like palmitoleic and oleic acids contribute approximately 10% to the total fatty acid content in both extracts (3% and 7%, respectively). The obtained results are consistent with earlier plant lipidomics research [34,35,36].

PLs rich in MUFAs and n-3 PUFAs, such as those structurally identified in the present study in the TAC extracts of both species, exert strong anti-PAF activities, with beneficial effects against several chronic disorders, including cardiovascular diseases, cancer and neurodegenerative disorders [2]. Therefore, it is safe to assume that the anti-PAF activity observed with the TAC extracts from both plants in the current study is a result of the presence of n-3 PUFAs and MUFA-rich PLs.

Such PLs have also been shown to exhibit potent anti-proliferative effects, particularly against colon cancer cells, through mechanisms involving apoptosis and growth inhibition [37]. Sphingolipids such as shingomyelins (SMs) are important in the nervous system, affecting neuronal growth, survival, and myelination while also influencing lipid metabolism and affecting insulin sensitivity [38]. Moreover, ceramides have been shown to have significant functions in early brain development and to be found in the brain in greater quantities than in many other organs [33]. Galactolipids like the DGDG identified in the TAC extracts of both plants have also been connected to the anti-inflammatory and anti-cancer advantages of a diet high in green, leafy vegetables in people [39], while SQDG, which was also identified in TAC extracts from both plants, is recognized for its significant biological activities, particularly in the context of human health, as it possesses notable antitumor and antiviral properties, attributed to its strong amphipathic character [40].

Overall, for all classes of polar lipids structurally identified in the present study, there exists a vast array of literature supporting their health-promoting properties against PAF, inflammation and associated disorders.

### 2.6. Analysis of Total Phenolics in TAC, TLC and TL Extracts from Lemna sp. and N. nucifera

The quantification of the total phenolic content (TPC) in all TAC, TLC and TL extracts from both *Lemna* sp. and *N. nucifera* was performed as previously described [29]. Results expressed in mg of gallic acid equivalent (GAE) per gram of dry weight (DW) of each extract are presented in Table 7.

Accordingly, a three-fold higher phenolic content was observed in the TAC extracts from *Lemna* sp. as opposed to those in the TLC of the same species (*p* < 0.05). In contrast, this difference was not observed between the phenolic content of TAC and TLC extracts in *N. nucifera*. Interestingly, *N. nucifera* seems to contain higher content of phenolic compounds than *Lemna* sp. in all TAC, TLC and TL extracts. The observed total phenolic content of *Lemna* sp. is comparable to that obtained in previous studies [9], which further support the antioxidant activity of this plant. Piya Temviriyanukul et al. [41] also reported that, for *N. nucifera*, the quantified phenolic content differed depending on the part of the plant analyzed. In the present study, the whole plant was chosen to be analyzed, in which the different phenolics from each part of the plant contributed to the overall phenolic content of the extracts, explaining why the total phenolic content detected in the present study is much higher than values previously reported for the individual contents of each part. Nevertheless, it seems that the rhizome contributes to the total phenolic content of the whole plant, as suggested by Jeong Soon You et al. [42].

### 2.7. Analysis of Total Carotenoids in TAC, TLC and TL Extracts from Lemna sp. and N. nucifera

The quantification of the total carotenoid content (TCC) in all TAC, TLC and TL extracts from both *Lemna* sp. and *N. nucifera* was performed as previously described [29]. Results are expressed in mg of beta-carotene equivalent (βCE) per gram of dry weight (DW) of each extract and are presented in Table 8.

TLC extracts showed higher carotenoid content than the TAC extracts in both aquatic plants. Interestingly, both TAC and TLC extracts from *Lemna* sp. showed higher TCC content than the corresponding extracts from *N. nucifera*. Furthermore, TL from *Lemna* sp. contains higher carotenoid content than the TL of *N. nucifera*. Significant amounts of beta-carotene and lycopene in *Lemna* sp. have been previously reported [43] as the major carotenoid compounds that contribute to the antioxidant activity of the plant. In addition, beta-carotene is a precursor of vitamin A, which has skin-rejuvenating effects, improves vision, and strengthens the immune system [44]. Of course, much higher amounts of carotenoids were detected in the present study, probably due to the isolation of many more types of compounds than just the two mentioned above. Regarding the efficiency of the present assay for the isolation of total carotenoids from *N. nucifera*, it was found that it results in the extraction of higher yields of these antioxidant bioactives compared to those derived from other extraction techniques [45].

More specifically, the part of the method that differs and most likely leads to the isolation of a higher percentage of carotenoids is that in the present study, the most optimum mixtures of organic solvents were selected and used for extraction of the total amphiphilic and lipophilic compounds, including in them the carotenoid contents of these plant sources. In contrast, in other extraction procedures, mostly hydroalcoholic mixtures of solvents were used for extraction of antioxidants. It seems that these more polar mixtures of solvents used in the other studies are not the optimum ones for co-extraction of carotenoids, polar lipids and phenolics within the same extract, as was achieved in the present study with the Bligh Dyer [11] extraction procedure and the Galanos and Kapoulas [12] countercurrent distribution methodology, as modified by Tsoupras et al. [13].

### 2.8. Antioxidant Activities of TAC, TLC and TL Extracts from Lemna sp. and N. nucifera

The evaluation of the antioxidant activity of all the TAC, TLC and TL extracts from both *Lemna* sp. and *N. nucifera* was carried out via three different assays—the 1,1-diphenyl-2-picrylhydrazyl (DPPH) radical scavenging assay, the ABTS (2,2′-azinobis-(3-ethylbenzothiazoline-6-sulfonic acid)) radical cation decolorization method and the ferric-reducing antioxidant power (FRAP) method—in comparison with a Trolox standard (water-soluble analog of vitamin E), as previously described [29].

In the DPPH assay, a free radical scavenger reacts with DPPH to form DPPH-H, which is less absorbent at a specific wavelength than DPPH due to its lower hydrogen content. Compared to the DPPH-H state, this version of the radical causes discoloration (yellowing) as the number of collected electrons increases. The ABTS assay measures free radical scavenging activity, i.e., the relative ability of antioxidants to remove ABTS produced in the aqueous phase. The FRAP assay measures the antioxidant potential of samples by the reduction of iron (Fe^3+^) to iron (Fe^2+^) provided by the antioxidants contained in the samples, i.e., an assay that measures the iron-reducing capacity of plasma. The antioxidant activity of all extracts from each plant shows differences between different TLC and TAC extracts, as well as within each test (Table 9 and Table 10).

More specifically, according to the results obtained from the DPPH-based assay (Table 9), the Trolox equivalent antioxidant capacity (TEAC) was only detected in the TAC extracts from both plants, while no antioxidant capacity was detected in the TLC from both plants by the DPPH methodology. These results suggest that only the more polar amphiphilic compounds (probably the phenolic compounds) of TAC were able to successfully participate in the DPPH-based reaction of this assay with positive detection. The TLC extracts, which contain lipophilic molecules like triglycerides, cholesterol esters, etc., i.e., richer in less polar carotenoids, were not able to provide a positive reaction in this assay due to being less soluble in the polar aquatic conditions of the assay solution.

It should also be mentioned that the TEAC value for an extract based in this assay is the ratio of the concentration of Trolox providing the half-maximum effect (IC_50_) over the IC_50_ of the extract with respect to its antioxidant activity of scavenging free radicals. This means that the higher the TEAC value for an extract, the lower its IC_50_ value and, thus, the higher its antioxidant capacity for the DPPH assay. Nevertheless, even though the TAC from *N. nucifera* showed slightly higher antioxidant capacity than the corresponding *Lemna* sp. extract, the TEAC values did not differ significantly.

The presence of DPPH antioxidant activity in both aquatic plants is in accordance with previously reported results. More specifically, the values obtained here in for *N. nucifera* are within the average of the reported results for various parts of the plant [46]. Regarding *Lemna* sp., its strong antioxidant activity against the DPPH reagent can be further enhanced when incorporated in gold nanoparticles, leading to drastic reductions in free radicals and oxidative stress in the studied models [47].

Regarding the antioxidant activity detected using the ABTS assay, the results (ABTS values) are expressed as μmol Trolox equivalent (TE) per g dry weight of the extract (Table 10).

As shown in Table 10, the results differ between the two aquatic plants. Specifically, ABTS values were only detected in the TLC extracts from *Lemna* sp., while no antioxidant capacity was detected in the TAC from this aquatic plant. In contrast, in *N. nucifera*, ABTS values were detected in both TAC and, in particular, TLC, contributing significantly to the total antioxidant capacity of the TL.

Concerning *Lemna* sp., the overall activity obtained based on the ABTS assay is lower than that observed in other studies [9]. It seems that the lower amounts of phenolics and carotenoids detected in the TAC extract were not able to provide a positive reaction. In contrast, the TLC, which contain more antioxidant carotenoids, showed antioxidant capacity, albeit low. On the other hand, the results obtained from this assay for Indian lotus were much higher in both TAC and TLC, and the overall ABTS-based antioxidant activities for this aquatic plant were found to be even better than those observed in previous studies [46]. It seems that the much higher phenolic contents in both TAC and TLC extracts of *N. nucifera* are responsible for this effect.

The antioxidant activities of all TAC, TLC and TL extracts from both plants were also evaluated using a FRAP assay, with the obtained results presented in Table 11. Antioxidant capacity, expressed as FRAP values, was detected in the extracts from Indian lotus, whereas no antioxidant activity was detected in any extract from *Lemna* sp.

Again, these differences in the antioxidant capacities of the two plants seem to be related to the phenolic contents present in their TAC and TLC extracts rather than to their carotenoid contents. It seems that the more polar phenolics tend to react better in the more polar aquatic conditions/solutions, in which these assays are performed, with the more lipophilic carotenoids having an apparently limited contribution in these assays. This is consistent with the much higher phenolic contents detected in all extracts of *N. nucifera*, resulting in considerable FRAP values in both TAC and TLC extracts. Moreover, the high overall antioxidant activity observed in *N. nucifera* by this assay is comparable to those obtained in other studies [46].

As compared to *Lemna* sp., the TAC, TLC and TL extracts of *N. nucifera* exhibited much higher antioxidant capacity in all three performed assays—especially in the ABTS and FRAP assays, which were performed under more polar conditions. This further indicates that the higher phenolic content of Indian lotus mainly contributes to these antioxidant activities for all extracts from this aquatic plant. In contrast, the lower phenolic content of *Lemna* sp. was not enough to provide any positive results for these assays, except in the case of the DPPH assay, in which some alcohol was added to dissolve the sample prior to the reaction with the DPPH aquatic solution. This seems to facilitate the contribution of not only the low phenolic content but also that of the higher carotenoid content observed in the TAC extract of this plant. Thus, only in the DPPH assay was some considerable antioxidant capacity detected for the TAC extracts of *Lemna* sp.

Nevertheless, in order to better understand the differences between the antioxidant activities of the extracts from these aquatic plants, more targeted tests are needed to evaluate their antioxidant capacities under both polar aquatic and polar amphiphilic/lipophilic conditions.

## 3. Materials and Methods

### 3.1. Sample Preparation and Extraction of Bioactive Compounds from the Aquatic Plants

#### 3.1.1. Materials—Instrumentation

The samples of *Lemna* sp., were collected from the last stage of biological water treatment facilities of Nea Peramos, Kavala, Greece. The sample of *N. nucifera* was commercially purchased from the Gryllis Water Lilies plant nursery (https://grylliswaterlilies.gr/, accessed 20 January 2024). All chemical reagents (Folin-Ciocalteu, DPPH and ABTS), solvents (chloroform, methanone, petroleum ether, ethanol, n-octane and isopropanol), phenolics (Trolox, gallic acid, quercetin and catechin) and lipid standards (soy polar lipids and beta-carotene) were purchased from Sigma Aldrich (St. Louis, MI, USA). UV-vis spectroscopy analyses were performed on an LLG-uniSPEC 2 spectrophotometer, and ATR-FTIR spectroscopy was performed on a Perkin Elmer Frontier ATR/FT-NIR/MIR spectrophotometer.

All plastic consumables, reagents and solvents used in the antiplatelet assays were of analytical grade and purchased from Sigma Aldrich. The 20-gauge (G) safety needles and evacuated sodium citrate S-monovettes^®^ used for blood collection were supplied by Sarstedt Ltd. (Wexford, Ireland). Human PRP (hPRP) bioassays were performed on a Chrono-log-490 four-channel strobilometric platelet aggregator (Havertown, PA, USA) connected to the AGGRO/LINK^®^8 software package. All platelet aggregation consumables and ADP were purchased from Chrono-log (Havertown, PA, USA). The standards for PAF and bovine serum albumin (BSA) were purchased from Sigma Aldrich (St. Louis, MI, USA). Centrifugation was performed in a Nahita blue centrifuge (Medibas+Low Speed Centrifuge).

#### 3.1.2. Sample Preparation and Extraction of Bioactive Amphiphilic Compounds

For sample preparation, the total available mass of each plant was divided into three equal parts of 100 g each. This was done by equally dividing all the different parts of the plants (leaves, flowers, rhizome, stamens and stem) into equal parts. The whole plant was used in order to obtain a comprehensive profile of total amphiphilic compounds, as literature data indicate that lipids and phenolics are distributed in all parts of the plant. Total lipids (TL) were extracted from each sample based on a modification of the Bligh and Dyer extraction method [11] according to Tsoupras et al. [13]. More specifically, after this separation, each sample was pulverized in a mortar with a small amount of a single-phase system containing chloroform/methanol/water in a ratio of 1:2:0.8 (*v*/*v*/*v*). Then, the massed plants were transferred to a glass beaker, and further volumes of this solvent were used to transfer the remnants from the mortar to the glass until the total volume within the glass reached 380 mL (approximately a 4/1 ratio between the volume of the solvent used in mL and the initial mass of the homogenized plant), in which further homogenization of the massed plants took place. In the case of Indian lotus, it was also necessary to use an Inox domestic-use blender for the crushing of the harder parts within this glass. Subsequently, each plant homogenate was vacuum-filtered from the precipitated residues in a simple filtering paper by pumping in a Buchner filtration apparatus (Buchner funnel).

The solute extracts were transferred to a separatory funnel to obtain the total lipid (TL) content of each sample. By adding appropriate volumes of water and chloroform, a biphasic system was then achieved, containing chloroform/methanol/water in a ratio of 1:1:0.9 (*v*/*v*/*v*), which, after stirring, facilitated a phase separation, with TL obtained in the lower chloroform-based phase. This phase was concentrated in round-bottom flasks and evaporated to dryness in a flash rotary evaporator at 37 °C under vacuum between 350 and 50 mbar, then redissolved in small volumes of a 1/1 (*v*/*v*) chloroform/methanol solution and transferred to a small, preweighed glass tube, in which it was evaporated under a stream of nitrogen. The obtained TL was then weighed and stored under nitrogen at −20 °C for a maximum of 8 weeks.

Based on a modification of the countercurrent partitioning method of Galanos and Kapoulas [12], the obtained TL extracts of all samples were then further separated into their total amphiphilic compound (TAC) and total lipophilic compound (TLC) fractions as described by Tsoupras et al. [13]. Pre-extracted solvents, pre-equilibrated petroleum ether and 87% ethanol in water were used to obtain TLC and TAC extracts. After several repetitions (maximum of 8 times), the completion of this method yielded TAC in the ethanol phase and TLC in the petroleum ether phase in a separation funnel. Both phases were collected in round-bottom flasks and evaporated to dryness on a rotary evaporator. The TAC extracts were then redissolved within the flasks in small volumes of a 1/1 (*v*/*v*) chloroform/methanol solution and transferred to a small, preweighed glass tube, while the same procedure was also followed for the TLC extracts, for which small volumes of petroleum ether were used for transfer to the preweighted glass. In both cases, the solvents were evaporated under a stream of nitrogen, and the obtained TAC and TLC extracts were weighed and stored under nitrogen at −20 °C until further analysis.

### 3.2. FT-IR-Based Structural Analysis of TAC Extracts from Lemna sp. and N. nucifera

ATR-FTIR was performed as previously described [48]. In addition to the standards, each extract (sample to be analyzed) was solubilized in a small amount of isopropanol, which was used as solvent. The sample was placed on the plate in an amount that covered the crystal; then, the detector tip was adjusted to touch the plate. Once in contact, a green line appeared on the force gauge, and the force applied by the rotating tower was increased until the spectrum displayed on the work surface was stabilized. The spectrum obtained for each sample was analyzed against a set of standard samples for which the same procedure was previously performed. These standard samples were quercetin, catechin, gallic acid, beta-carotene and polar lipids from soy. At the same time, the spectrum of isopropanol was also analyzed in order to reduce any error in the study that may have been derived due to solvent interference with the sample under study. The peaks that were captured in the table are those with the highest intensity that appear in both the reference samples and the plant extracts.

### 3.3. Evaluation of Antithrombotic and Anti-Inflammatory Properties of the Extracts

Bioassays to evaluate the antiplatelet and anti-inflammatory properties of all TLC and TAC extracts from all plant samples were performed on human platelet-rich plasma (hPRP) specimens isolated from healthy donors (N = 6). Inhibition of PAF- and ADP-induced platelet aggregation in hPRP by the plant extracts was performed according to the method of Tsoupras et al. [13]. The anti-inflammatory and antithrombotic activity of each sample were expressed as the mean of the IC_50_ values (half-maximal inhibitory concentration) ± standard deviation (SD), quantified as the mass (μg) of the bioactive TAC or TLC extract present in the aggregometer cuvette that can cause a 50% inhibition of hPRP aggregation induced either by PAF (anti-PAF anti-inflammatory potency) or ADP (anti-ADP, anti-platelet effects). Each sample was evaluated several times on blood samples from different human volunteers (N = 6) to ensure reproducibility.

### 3.4. Evaluation of Fatty Acid Composition by LC-MS

Liquid chromatography–mass spectrometry (LC-MS) was used to evaluate the fatty acid composition of all TLC and TAC extracts from all plant samples, as described by Vandorou et al. [14]. Briefly, each dried extract was redissolved in 500 μL dichloromethane/methanol (1:2, *v*/*v*), then centrifuged at 13,000 rpm for 6 min (Heraeus Biofuge Stratos, Fisher Scientific Ltd., Dublin, Ireland). Filtration of the clear supernatants was performed using 3 kDa ultracentrifugation filters (Amicon Ultra 3 k, Merck Millipore Ltd., Darmstadt, Germany). To obtain fatty acid profiles in these filtrates, 10 μL of each filtrate was injected into an HPLC system (Agilent 1260 series, Agilent Technologies Ireland Ltd., Little Island, Co., Cork, Ireland) equipped with a Q-TOF mass spectrometer (Agilent 6520) using electrospray ionization (ESI) as the source type. Fatty acid separation was performed using an Agilent C18 Poroshell 120 column (2.7 µm, 3.0 × 150 mm) with a gradient elution in which mobile phase A consisted of 2 mM ammonium acetate in water, while mobile phase B consisted of 2 mM ammonium acetate in 95% acetonitrile. The mobile-phase flow rate was initially set at 0.3 mL/min for 5 min, increased to 0.6 mL/min after 10 min, and maintained at that flow rate until the end of the run. The mass spectrometer scanned from 50 to 1100 *m*/*z* while using reference masses of 1033.988 and 112.9855 to monitor the scan in negative ionization mode. The capillary voltage was 3500 V, and the scatterer and fragmenter voltages were maintained at 65 V and 175 V, respectively. The drying gas flow, pressure and nebulizer temperature were set at 5 L/min, 30 psi and 325 °C, respectively.

The validation of the LC-MS method was performed by comparing the obtained specific mass (molecular weight) and retention time (RT) of different standard saturated and unsaturated fatty acids, namely lauric (C12:0), myristic (C14:0), palmitic (C16:0), steatic (C18:0), oleic (OA, C18: 1n−9 cis), linoleic (LA, C18: 2n−6 cis), gamma−linolenic (GLA, C18: 3n−6), α−linolenic (ALA, C18: 3n−3), arachidonic (ARA, C20: 4n−6), eicosapentaenoic (EPA, 20:5n−3), docosapentaenoic (DPA, 22:5n−3) and docosahexaenoic (DHA, 22:6n−3) acids (Sigma, Ireland). The standards also facilitated the evaluation of each lipid extract sample in which each of its fatty acids was identified by its known accurate mass (molecular weight). The peak area of each identified fatty acid was the average of the three samples, and their relative content was recorded according to their average peak area. Since the peak areas do not reflect the exact amounts of individual fatty acids, the corresponding data should be read with caution.

The assignment of fatty acids and phospholipid species was based upon a combination of survey, daughter, precursor and neutral loss scans, and the identity of the bioactive PL molecules was verified using the LIPID MAPS: Nature Lipidomics Gateway (www.lipidmaps.org, accessed on 15 March 2025), with the lowest delta values combined with the results obtained from the LC-MS analysis on the fatty acid composition of the saponified PL, as previously described by Vandorou et al. [14].

### 3.5. Assesment of Total Phenolic and Carotenoid Content and Antioxidant Activity

#### 3.5.1. Preparation of Samples for Further Analysis

Each dry TAC sample was dissolved in 1 mL of ethanol, while each dry TLC sample was dissolved in 1 mL of octane. The total volume (1 mL) of each sample was aliquoted into individual equal volumes to perform the following tests in order to evaluate its bioactive components. Furthermore, small amounts of solvents were evaporated to obtain dry TAC and TLC samples in each aliquot for each analysis.

#### 3.5.2. Total Phenolic Content (TPC) Analysis

The phenolic content of all TLC and TAC extracts of each plant was evaluated using Folin–Ciocalteu reagent according to Papadopoulou et al. [29]. Specifically, 1 mL of distilled water and 1 mL of Folin–Ciocalteu reagent were added to each sample. After 7 min, an additional 3 mL of Na_2_CO_3_ was added to each sample. The samples were then incubated in the dark for 2 h. The solutions were stirred by vortexing between successive additions of reagents and every 30 min during the incubation. After two hours of incubation, the absorbance of the samples was measured at 765 nm. The required concentration was determined from the standard gallic acid curve, and the results are expressed as mg gallic acid equivalents (GAE)/g extract.

#### 3.5.3. Analysis of Total Carotenoid Content (TCC)

The quantification of the total carotenoid content (TCC) of each extract was performed according to Papadopoulou et al. [29]. More specifically, each sample was dissolved in 2 mL of octane, and the absorbance was measured at 450 nm. Samples with absorbances of more than 0.9 in the first measurement were diluted 1:2, and the measurement was repeated. This procedure was repeated until the absorbance of the samples was less than 0.8. The required concentration was measured according to the standard beta-carotene curve, and the obtained results are expressed as mg beta-carotene equivalents (CE)/g extract.

#### 3.5.4. Analysis of Total Antioxidant Activity

The evaluation of the antioxidant activity of the samples was carried out by the 1,1-diphenyl-2-picrylhydrazyl (DPPH) radical binding method, the 2,2′-azino-bis-(3-ethylbenzothiazolin-6-sulfonic acid) (ABTS) cationic radical binding assay and the trivalent iron reduction antioxidant power (FRAP) method according to Papadopoulou et al. [29].

For the DPPH method, 0.2 mL ethanol, 0.8 mL Tris-HCl buffer (pH 7.4) and 1 mL DPPH solution were added to each sample. The solutions were stirred with a vortex between successive additions of reagents. The solutions were kept at room temperature for 30 min, and the absorbance was recorded at 517 nm immediately thereafter.

The inhibition percentage (%) was calculated using the following equation:

Inhibition (%) = (A1 − A2) × 100∕A1,

where A1 is the absorbance of the control sample solution and A2 is the absorbance of the test sample solution.

The IC_50_, i.e., the concentration of each extract capable of neutralizing 50% of the DPPH radical, was then calculated. The DPPH radical scavenging activity of the sample is expressed as Trolox equivalent antioxidant capacity (TEAC). TEAC was calculated as follows:

TEAC = IC_50_ of Trolox (μg∕L)∕IC_50_ of the sample (μg∕L).

For the ABTS method, 2 mL of ABTS solution was added to each sample, followed by vortex stirring. The solutions were incubated in the dark for 7 min, and the absorbance was measured immediately at 734 nm. Trolox was used as the standard. The concentration of Trolox was selected so that the absorbance value was between 0.2 and 0.8 to construct a standard curve.

The result is expressed as μmol TE/g DW according to the following formula:

ABTS (μmol TE∕g DW) = c × V × t∕m,

where c is the Trolox concentration (µmol/mL) of the corresponding standard curve of the diluted sample, V is the volume of the sample (mL), t is the dilution factor and m is the dry weight of the sample (g).

For the FRAP method, 3 mL of FRAP solution was added to each sample, followed by vortex stirring. The solutions were incubated in the dark at 37 °C for 15 min, and the absorbance was immediately measured at 593 nm. Trolox was used as the standard. The concentration of Trolox was selected so that the absorbance value was between 0.2 and 0.8 to obtain a standard curve.

The result is expressed as μmol TE/g DW according to the following formula:

FRAP (μmol TE∕g DW) = c × V × t∕m,

where c is the Trolox concentration (µmol/mL) of the corresponding standard curve of the diluted sample, V is the volume of the sample (mL), t is the dilution factor and m is the weight of the dry substance of the sample (g).

### 3.6. Statistical Analysis of Results

The distributional characteristics of all measured parameters were assessed using the Kolmogorov–Smirnov test to determine adherence to normality assumptions. Parameters exhibiting a normal distribution were subjected to comparative analysis using one-way analysis of variance (ANOVA), allowing for the evaluation of intergroup differences under parametric conditions. For parameters deviated from normality, the Kruskal–Wallis test was employed as a nonparametric alternative to account for distributional nonconformity. A threshold of *p* < 0.05 was established as the criterion for statistical significance in all comparative analyses.

## 4. Conclusions

In the present study, *Lemna* sp. and *N. nucifera* extracts enriched with TAC bioactive compounds (polar lipids rich in unsaturated fatty acids, carotenoids and phenolics) were successfully produced.

These TAC bioactive compounds were found to possess potent anti-inflammatory properties, mainly against inflammatory and thrombotic mediator PAF, in addition to significant antiplatelet and, thus, antithrombotic benefits against the pathways of a standard platelet agonist, ADP, in human platelets. In addition, their high n-3 PUFA content and favorably low n-6/n-3 PUFA ratio values further support their anti-inflammatory potency.

Apart from their potent anti-inflammatory and antithrombotic properties, the amphiphilic compounds present in the TAC extracts also possess considerable antioxidant activities, especially those from *N. nucifera*, effectively neutralizing free radicals.

Therefore, it seems that TAC extracts from both plants—especially those from *Lemna* sp.—have a dual medicinal use, with both anti-inflammatory effects against PAF and anti-platelet effects against ADP, as well as potent antioxidant capacity. However, further analyses of their activity under different analytical conditions are needed. Moreover, the initial results of the present study on the potent antioxidant, anti-inflammatory and antithrombotic biological activities of the TAC extracts of these two aquatic plants open new avenues of research for their sustainable exploitation for the production of new biofunctional products with cardioprotective properties and preventive potential against oxidative stress and chronic inflammation-related disorders, including cancer.

Overall, the present study highlights the potential of *Lemna* sp. and *N. nucifera* as valuable natural sources of TAC to be valorized as bioactive ingredients in several functional products with therapeutic properties, with potential applications in combating chronic diseases through the development new functional products, like food supplements, functional foods, and cosmetic and pharmaceutical products with potent anti-inflammatory and antithrombotic activities and associated health-promoting properties.

However, further research is needed to validate these findings through clinical trials in order to fully evaluate the in vivo safety and efficacy of the antioxidant and anti-inflammatory properties for functional products containing bioactive TAC ingredients from these aquatic plants.

## Figures and Tables

**Table 1:** Yields of TL, TLC and TAC extracts from *Lemna* sp. and *N. nucifera* samples.

Sample	TAC *	TLC *	TL *
*Lemna* sp.	0.0502 ± 0.0101	0.0281 ± 0.0089	0.0783 ± 0.0190
*N. nucifera*	0.0513 ± 0.0317	0.0278 ± 0.0277	0.0791 ± 0.0586

* Results are expressed as means of g of extract/100 g of sample ± standard deviation (abbreviations: TAC = total amphiphilic compounds; TLC = total lipophilic compounds; TL = total lipids.)

**Table 2:** Characteristic FT-IR absorption peaks for TAC extracts from *Lemna* sp. with corresponding functional group assignments and comparison with standard solutions.

Number of Peaks	Transmittance Τ%	Wavenumber (cm^−1^)	IR Vibration	Similarities with Standard Solutions
1	86.19	3300–3400	OH stretching	Galic Acid, Quercetin, Catechin
2	77.39	2800–2900	C-H stretching	b-Carotene, Polar Lipids
3	84.92	1450–1500	-Ph ring	Galic Acid, Quercetin, Catechin
4	81.04	1300–1450	C-H bending	b-Carotene, Polar Lipids
5	79.54	1087–1124	C-O-C stretching	Quercetin, Catechin
6	78.76	900–950	=C-H bending	b-Carotene
7	87.18	800–850	C-H bending	Catechin

**Table 3:** Characteristic FT-IR absorption peaks for TAC extracts from *N. nucifera* with corresponding functional group assignments and comparison with standard solutions.

Number of Peaks	Transmittance Τ%	Wavenumber (cm^−1^)	IR Vibration	Similarities with Standard Solutions
1	89.11	2924	C-H stretching	b-Carotene, Polar Lipids
2	87.77	1726–1728	C=O stretching of phenolic group	Galic Acid, Quercetin
3	86.6	1440–1442	C-H bending	b-Carotene, Polar Lipids
4	84.52	1228–1234	C-O stretching	Galic Acid, Quercetin, Catechin
5	77.95	1060	C-O-C bending	Catechin

**Table 4:** The profile of esterified fatty acids of TAC extracts for each aquatic plant, expressed for each FA as the mean value of the % of total fatty acids of each evaluated sample (mean ± standard deviation (SD), n = 3).

Fatty Acid Empirical Name	Fatty Acid Name	*Lemna* sp.	*N. nucifera*
Caprylic	C8:0	ND	ND
Pelargonic	C9:0	0.09 ± 0.02	0.12 ± 0.01
Capric	C10:0	ND	ND
Lauric	C12:0	0.13 ± 0.01	0.06 ± 0.0
Tridecylic	C13:0	ND	ND
Myristic	C14:0	0.61 ± 0.01	0.46 ± 0.02
Pentadecylic	C15:0	0.46 ± 0.08	0.64 ± 0.01
Palmitic	C16:0	20.74 ± 4.23	22.01 ± 0.46
Palmitoleic	C16:1 c9 (n7 MUFA)	2.16 ± 0.15	2.51 ± 0.02
Margaric	C17:0	0.66 ± 0.25	0.62 ± 0.03
Stearic	C18:0	12.21 ± 2.32	9.17 ± 0.63
Oleic	C18:1 c9 (n9 MUFA)	7.07 ± 2.01	5.86 ± 0.23
Linoleic	C18:2 c9,12 (n6 PUFA)	11.17 ± 1.21	14.87 ± 0.21
Linolenic (α + γ)	C18:3 c9,12,15 (n3 PUFA)	33.75 ± 4.76	40.90 ± 0.40
Stearidonic	C18:4 c6,9,12,15 (n3 PUFA)	2.32 ± 0.21	0.27 ± 0.0
Nonadecylic	C19:0	ND	ND
Arachidic	C20:0	ND	ND
Gadoleic	C20:1 c9 (n11 MUFA)	0.42 ± 0.10	0.37 ± 0.01
DihomoLinoleic	C18:2 c10,12 (n6 PUFA)	0.86 ± 0.24	0.27 ± 0.01
Dihomolinolenic	C20:3 c8,11,14 (n6 PUFA)	0.40 ± 0.08	0.25 ± 0.01
Arachidonic	C20:4 c5,8,11,14 (n6 PUFA)	0.79 ± 0.04	0.65 ± 0.01
EPA	C20:5 c5,8,11,14,17 (n3 PUFA)	2.50 ± 0.35	0.53 ± 0.01
Docosadienoic	C22:2 c13,16 (n6 PUFA)	ND	ND
Eranthic	C22:3 c5,13,16 (n6 PUFA)	0.25 ± 0.10	ND
Adrenic	C22:4 c7,10,13,16 (n6 PUFA)	ND	0.10 ± 0.01
DPA	C22:5 c7,10,13,16,19 (n3 PUFA)	0.26 ± 0.04	0.18 ± 0.0
DHA	C22:6 c4,7,10,13,16,19 (n3 PUFA)	3.14 ± 0.62	0.17 ± 0.0
	**SFA**	34.91 ± 2.30	33.08 ± 0.66
	**UFA**	65.09 ± 2.30 *	66.92 ± 0.66 *
	**MUFA**	9.65 ± 2.05	8.74 ± 0.24
	**PUFA**	55.44 ± 4.32 **	58.18 ± 0.64 **
	**n3PUFA**	41.97 ± 5.89	42.04 ± 0.41
	**n6PUFA**	13.47 ± 1.59	16.14 ± 0.23
	**n6/n3**	0.33 ± 0.08	0.38 ± 0.0

* and ** denote statistically significant differences (*p* < 0.05) between SFA with UFA and PUFA with MUFA and SFA, respectively, for each plant.

**Table 5:** The free fatty acid profile of TAC extracts for each aquatic plant, expressed for each FA as the mean of the % of total fatty acids of each evaluated sample (mean ± standard deviation (SD), n = 3).

Fatty Acid Empirical Name	Fatty Acid Name	*Lemna* sp.	*N. nucifera*
Caprylic	C8:0	ND	ND
Pelargonic	C9:0	0.36 ± 0.01	0.51 ± 0.03
Lauric	C12:0	ND	ND
Tridecylic	C13:0	ND	ND
Myristic	C14:0	1.31 ± 0.03	1.04 ± 0.12
Pentadecylic	C15:0	0.92 ± 0.11	1.10 ± 0.18
Palmitic	C16:0	25.65 ± 1.14	37.94 ± 1.40
Palmitoleic	C16:1 c9 (n7 MUFA)	2.71 ± 0.09	2.78 ± 0.14
Margaric	C17:0	1.75 ± 0.02	ND
Stearic	C18:0	44.23 ± 1.32	43.55 ± 2.50
Oleic	C18:1 c9 (n9 MUFA)	7.02 ± 0.35	7.11 ± 0.62
Linoleic	C18:2 c9,12 (n6 PUFA)	6.76 ± 0.11	3.19 ± 0.21
Linolenic (α + γ)	C18:3 c9,12,15 (n3 PUFA)	4.56 ± 0.12	2.53 ± 0.25
Stearidonic	C18:4 c6,9,12,15 (n3 PUFA)	0.43 ± 0.02	ND
Nonadecylic	C19:0	ND	ND
Gadoleic	C20:1 c9 (n11 MUFA)	ND	ND
Dihomolinoleic	C18:2 c10,12 (n6 PUFA)	0.8 ± 0.02	ND
Dihomolinolenic	C20:3 c8,11,14 (n6 PUFA)	0.31 ± 0.02	ND
Arachidonic	C20:4 c5,8,11,14 (n6 PUFA)	0.42 ± 0.0	ND
EPA	C20:5 c5,8,11,14,17 (n3 PUFA)	1.21 ± 0.04	0.25 ± 0.08
Docosadienoic	C22:2 c13,16 (n6 PUFA)	ND	ND
Eranthic	C22:3 c5,13,16 (n6 PUFA)	ND	ND
Adrenic	C22:4 c7,10,13,16 (n6 PUFA)	ND	ND
DPA	C22:5 c7,10,13,16,19 (n3 PUFA)	ND	ND
DHA	C22:6 c4,7,10,13,16,19 (n3 PUFA)	1.54 ± 0.05	ND
	**SFA**	74.23 ± 2.63 *	84.14 ± 4.24 *
	**UFA**	25.77 ± 0.82 **	15.86 ± 1.31 **
	**MUFA**	9.73 ± 0.44	9.89 ± 0.77
	**PUFA**	16.04 ± 0.38	5.97 ± 0.54
	**n3PUFA**	7.75 ± 0.23	2.78 ± 0.33
	**n6PUFA**	8.29 ± 0.15	3.19 ± 0.21
	**n6/n3**	1.07 ± 0.65	1.15 ± 0.65

* indicates a statistically significant difference (*p* < 0.05) between SFA and UFA for each plant; ** indicates a statistically significant difference (*p* < 0.05) between *Lemna* sp. UFA and *N. nucifera* UFA.

**Table 6:** Representative molecular species of the main classes of polar lipid bioactive compounds detected in TAC extracts of *Lemna* sp. and *N. nucifera* by LC–MS analysis.

	*Lemna* sp.				*N. nucifera*			
	Elution Time (min)	MS	Species	Representative Examples	Elution Time (min)	MS	Species	Representative Examples
**PC**	12.5–13.5	792.8612	PC 38:5	[i.e., PC 18:0 + 20:5 or PC 18:1 + 20:4]				
	12.5–13.5	792.8612	PC O-38:6; O	[i.e., PC O- 16:0 + 22:6; O or PC O- 18:1 + 20:5; O or PC O- 18:2 + 20:4; O]				
**PA**	2–3	675.3589	PA O-36:7	[i.e., PA O-18:3 + 18:4]				
	2–3	675.3589	PA 34:0	[i.e., PA 18:0 + 16:0]				
	2–3	675.3589	PA O-34:1; O	[i.e., PA O- 18:1 + 16:0; O or PA O- 18:0 + 16:1; O]				
**PG**	11.5–12-5	815.4935	PG 40:9	[i.e., PG 20:4 + 20:5]	12–13	815.4953	PG 40:9	[i.e., PG 20:4 + 20:5;]
**PI**	11.5–12.5	815.4935	PI O-34:4	[i.e., PI O-18:3 + 16:1 or PI O-18:4 + 16:0]	12–13	815.4953	PI O-34:4	[i.e., PI O-18:3 + 16:1; or PI O-18:4 + 16:0;]
**PE**					10–11 10–11 10–11	744.4135 744.4135 744.4135	PE O-38:8 PE 36:1 PE O-36:2; O	[i.e., PE O-18:3 + 20:5; or PE O-18:4 + 20:4;] [i.e., PE 18:1 + 18:0;] [i.e., PE O-18:2 + 18:0; O or PE O- 18:1 + 18:1; O]
**PS**	12.5–13.5 7.8–9.3	792.8612 792.8509	PS O-36:0; O PS O-38:6	[i.e., PS O-18:0 + 18:0; O] [i.e., PS O-16:0 + 22:6; or PS O-18:1 + 20:5; O or PS O- 18:2 + 20:4; O]	10–11	828.3959	PS 40:9	[i.e., PS 20:4 + 20:5;]
**ICP**	7.8–9.3	792.8509	IPC 34:2; O3	[i.e., IPC 18:1 + 16:1; O3 or IPC 18:2 + 16:0; O3]	10–11 10–11	828.3959 828.3959	IPC 36:6; O4 IPC 34:0;O5	[i.e., IPC 18:4 + 18:2; O4 or IPC 16:1 + 20:5; O4] [i.e., IPC18:0 + 16:0; O5]
**DGDG**					8.5–9.5 8.5–9.5	933.6881 966.0005	DGDG 36:7 DGDG 38:5	[i.e., DGDG 18:3 + 18:4;] [i.e., DGDG 18:0 + 20:5; or DGDG 18:1 + 20:4;]
**CerPE**	2–3	675.3589	CerPE 34:1; O3	[i.e., CerPE 18:1 + 16:0; O3 or CerPE 18:0 + 16:1; O3]	2–3	721.3534	CerPE 34:2; O6	[i.e., CerPE 18:1 + 16:1; O6 or CerPE 18:2 + 16:0; O6]
**Cer**	10–11	520.908	Cer 34:1; O	[i.e., Cer 18:1 + 16:0; O or Cer 18:0 + 16:1; O]	10–11	555.9965	Cer 36:5; O2	[i.e., Cer 18:3 + 18:2; O2 or Cer 18:4 + 18:1; O2 or Cer 16:0 + 20:5; O2 or Cer 16:1 + 20:4; O2]
**CerP**	1–2	656.8805	CerP 36:3; O3	[i.e., CerP 18:3 + 18:0; O3 or CerP 18:2 + 18:1; O3]	1–2	666.0196	CerP 36:6; O4	[i.e., CerP 18:4 + 18:2; O4 or CerP 16:1 + 20:5; O4]
**SM**	2–3	675.3589	SM 32:1; O3	[i.e., SM 16:0 + 16:1; O3]	6–6.5 12–13	734.0131 761.5929	SM 36:0; O3 SM 36:2; O5	[i.e., SM 18:0 + 18:0; O3] [i.e., SM 18:2 + 18:0; O5 or SM 18:1 + 18:1; O5]
**SQDG**	11.5–12.5	815.4935	SQDG 34:3	[i.e., SQDG 18:3 + 16:0; or SQDG 18:2 + 16:1;]	12–13	815.4953	SQDG 34:3	[i.e., SQDG 18:3 + 16:0; or SQDG 18:2 + 16;]
**MIPC**	7.8–9.3	928.8225	MIPC 32:1; O3	[i.e., MIPC 16:0 + 16:1; O3]				
**HexCer**	10–11	792.8567	HexCer 36:0; O6	[i.e., HexCer 18:0 + 18:0; O6]	10–11	744.4135	HexCer 34:2; O5	[i.e., HexCer 18:1 + 16:1; O5 or HexCer 18:2 + 16:0; O5]
**SHexCer**	12.5–13.5	792.8509	SHexCer 34:2; O3	[i.e., SHexCer 18:1 + 16:1; O3 or SHexCer 18:2 + 16:0; O3]	10–11	828.3959	SHexCer 36:6; O4	[i.e., SHexCer 18:4 + 18:2; O4 or SHexCer 16:1 + 20:5; O4]

**Table 7:** Total phenolic content (TPC) of TAC, TLC and TL extracts from *Lemna* sp. and *N. Nucifera*. Results are expressed as mg GAE/g DW of extract.

*Lemna* sp.	Median	Min	Max
**TAC**	16.476 *	12.656	18.294
**TLC**	5.085	3.935	5.557
**TL**	21.561	16.592	23.851
***N. nucifera***	**Median**	**Min**	**Max**
**TAC**	46.002 **	29.776	93.879
**TLC**	56.170	10.557	73.524
**TL**	102.172	40.333	167.403

* indicates a statistically significant difference of the TPC of TAC versus that of TLC in *Lemna* sp. (*p* < 0.05). ** indicates a statistically significant difference of the TPC of TAC from *N. nucifera* versus that of TAC from *Lemna* sp. (*p* < 0.05). Abbreviations: TL = total lipids; TAC = total amphiphilic compounds; TLC = total lipophilic compounds; TPC = total phenolic content; GAE = gallic acid equivalent; DW = dry weight.

**Table 8:** Total carotenoid content (TCC) of TAC, TLC and TL extracts from *Lemna* sp. and *N. nucifera*. Results are expressed as mg β-CE/g DW of extract.

*Lemna* sp.	Median	Min	Max
**TAC**	27.031 *	13.791	37.811
**TLC**	78.104 *	33.430	121.820
**TL**	115.915 *	47.221	148.851
***N. nucifera***	**Median**	**Min**	**Max**
**TAC**	5.624 *	3.782	15.735
**TLC**	7.593 *	6.388	22.115
**TL**	13.217 *	10.171	37.851

* indicates a statistically significant difference of the TCC of TAC, TLC and TL from *Lemna* sp. versus the TCC for each extract from *N. nucifera* (*p* < 0.05). Abbreviations: TL = total lipids; TAC = total amphiphilic compounds; TLC = total lipophilic compounds; TCC = total carotenoid content; β-CE = beta-carotene equivalent; DW = dry weight.

**Table 9:** Antioxidant capacity (AC) of the TAC, TLC and TL extracts from *Lemna* sp. and *N. nucifera*. Results are expressed as Trolox equivalent antioxidant capacity (TEAC) values according to the DPPH-based assay.

*Lemna* sp.	Median	Min	Max
**TAC**	0.780	0.294	1.664
**TLC**	ND		
**TL**	0.780	0.294	1.664
***N. nucifera***	**Median**	**Min**	**Max**
**TAC**	1.090	0.237	2.085
**TLC**	ND		
**TL**	1.090	0.237	2.085

Abbreviations: TL = total lipids; TAC = total amphiphilic compounds; TLC = total lipophilic compounds; TEAC = Trolox equivalent antioxidant capacity; ND = not detected.

**Table 10:** Antioxidant capacity (AC) of the TAC, TLC and TL extracts from *Lemna* sp. and *N. nucifera* according to the ABTS-based assay. Results are expressed as ABTS values (μmol TE/g DW of extract).

*Lemna* sp.	Median	Min	Max
**TAC**	ND		
**TLC**	2.568	1.906	3.948
**TL**	2.568	1.906	3.948
***N. nucifera***	**Median**	**Min**	**Max**
**TAC**	6.560	0.042	12.124
**TLC**	15.290	1.439	19.514
**TL**	26.074	1.481	27.414

Abbreviations: TL = total lipids; TAC = total amphiphilic compounds; TLC = total lipophilic compounds; TE = Trolox equivalent; DW = dry weight; ND = not detected.

**Table 11:** Antioxidant capacity (AC) of the TAC, TLC and TL extracts from *Lemna* sp. and *N. Nucifera* according to the FRAP-based assay. Results are expressed as FRAP values (μmol TE/g DW of extract).

*Lemna* sp.	Median	Min	Max
**TAC**	ND		
**TLC**	ND		
**TL**	ND		
***N. nucifera***	**Median**	**Min**	**Max**
**TAC**	60.991	35.205	94.936
**TLC**	11.109	1.707	70.302
**TL**	72.100	36.912	165.238

Abbreviations: TL = total lipids; TAC = total amphiphilic compounds; TLC = total lipophilic compounds; TE = Trolox equivalent; DW = dry weight; ND = not detected.

## Data Availability

All data are contained within the article. Any further information concerning raw data (i.e., FT-IR spectra and LC–MS chromatograms and spectra) can be provided by the authors upon request.
